# Supplementary material for: Stroke-related length of hospitalization trends and in-hospital mortality in Peru
Source: PeerJ. 2022 Nov 25;10:e14467. doi: 10.7717/peerj.14467 (PMC9703986; doi:10.7717/peerj.14467)
Supplement: Supplemental Information 1 [file peerj-10-14467-s001.docx]

**Supplemental methods [21]**

This study is a retrospective cohort, which used health registries of the Ministry of Health. Patients with stroke were treated in first, second, and third-level facilities of the Ministry of Health in the period 2002-2017. Patients with stroke who were seen and discharged by physicians had a single ICD-10 code diagnosis.

The stroke registries were included according to whether they met the inclusion or exclusion criteria. Between the inclusion criteria were: hospital registries that have a single diagnosis with an ICD-10 code, registries of stroke patients aged 35 years or older, hospital registries that included a single primary diagnosis of subarachnoid hemorrhage (I60), intracerebral hemorrhage (I61), cerebral infarction (I63), and stroke not identified as hemorrhagic or ischemic (I64), and registries of stroke patients who were seen and discharged by physicians. The exclusion criteria were registries of patients whose discharge was due to voluntary withdrawal, referral to another health facility, and absconding (those patients who left the hospital without making payments for administrative procedures), and cases of stroke with extreme values of hospital stay, above and below the 1st and 99th percentiles.

Exposure variables: To describe trends in hospital stay from 2002 to 2017 and hospital mortality from 2016 to 2017, sociodemographic and clinical variables were used: gender; age (35-54, 55-74 and ≥75 years); region of Peru where the hospitalization occurred (Lima / Callao, rest of the Coast (included Tumbes, Piura, Lambayeque, La Libertad, Ancash, Ica, Arequipa, Moquegua, Tacna), Amazonia (included Amazonas, San Martin, Loreto, Ucayali, Madre de Dios), Sierra (included Cajamarca, Huánuco, Pasco, Junín, Huancavelica, Ayacucho, Apurímac, Cusco, Puno)); level of the health facility where the hospitalization occurred (level I: primary care facilities; level II: intermediate care facilities; and level III: hospitals and specialized institutes); and type of stroke (ICD-10: subarachnoid hemorrhage (I60), intracerebral hemorrhage (I61), cerebral infarction (I63) used as a control or reference group, and stroke not specified as hemorrhage or infarction (I64)). In the analysis of in-hospital mortality, the type of stroke was the exposure of interest. The variables sex and age were used to adjust the analysis.

Outcome variables: i) Length of stay (in days): defined as the difference between discharge date and hospitalization date; for this outcome, data from 2002 to 2017 was used. ii) In-hospital mortality: a time-event variable, defined as the time, in days, elapsed from hospital admission until the stroke case was discharged for the medical indication (censored) or died during hospitalization. For survival analysis, all data, time to death or discharge, were included.

Methods of data collection: A retrospective cohort study was conducted using secondary data: open access information from the registries of the Ministry of Health, through a request through its website: <http://www.minsa.gob.pe/portada/transparencia/solicitud/frmformulario.asp>. The aggregate registries were accessed and the variables of interest corresponding to the health center, health center level, region, sex, patient age, type of stroke, mortality, and hospital admission and discharge date were selected. All stroke registries were included according to whether they met the inclusion or exclusion criteria. Two databases were generated, according to the availability of the variables of interest: a. Length of stay data were available during the period 2002-2017. b. Mortality data were only available for the period 2016-2017.

Statistical methods: For the descriptive analysis of the numerical variables, the assumptions of normality were evaluated, using the Shapiro-Wilk test, according to which the best measure of central tendency and dispersion for the numerical variables was selected; in the case of categorical variables, frequencies and percentages were described. Variables were described using mean/median or absolute/relative frequencies, depending on distribution and whether they were numerical or categorical. The Kolmogorov-Smirnov test was used to determine the non-normal distribution of the variables; Hospital stay was identified as a non-normal variable and medians were calculated along with interquartile ranges (IQR). Comparisons for categorical variables were made using the Chi2 test, while the Kruskal-Wallis test was used for numerical variables, corrected for multiple comparisons (Dunn's test with Bonferroni correction). The Mann-Whitney-Wilcoxon rank sum test was used for the comparison between two numerical variables. To evaluate the trend of sex, it was estimated with the correlation trend test with Spearman's correlation coefficients (rho). The case fatality rate (LF) was calculated considering the Poisson distribution with 95% confidence intervals (95% CI). To obtain p-values, the two-proportion z-test was used. Regarding the analysis of in-hospital mortality, hospital survival by stroke subtypes was examined, using the Kaplan-Meier method (unadjusted) and the log-rank test. In addition, a Weibull proportional hazard model stratified by region and hospital was fitted, after proportional hazard assumptions for primary exposure as well as additional covariates (age and sex) had been tested. The likelihood ratio test was used to assess the inclusion of interactions in the Weibull model stratified by region and hospital level: stroke type and health facility level, as well as stroke type and regions. In addition, the proportional hazard assumption was corroborated using the Schoenfeld test, and the Weibull distribution of baseline risk was assessed using log(-log(survival)) and log(time) plots. The analyzes were performed with the free software R (v4.0.0) (62) and Stata 15.0 (Stata Corp, College Station, TX, USA). The analysis code is available on the GitHub platform: <https://github.com/llabans/stroke>.
